# New Method for Measuring Statistical Distributions of Partial Discharge Pulses

**DOI:** 10.6028/jres.102.038

**Published:** 1997

**Authors:** Yicheng Wang

**Affiliations:** National Institute of Standards and Technology, Gaithersburg, MD 20899-0001

**Keywords:** amplitude distributions, log-normal distribution, partial discharges, random point process, time separation distributions

## Abstract

A new digital detection system is described for measuring pulsating partial discharges (PDs). The PD detection system can continuously record all PD pulses that occur over extended periods of time, with a minimum inter-pulse time separation of 6 μs and a vertical amplitude resolution of 12 bits. Earlier PD detection systems detected PD pulse amplitude and time using complex custom-designed hardware while the present system continuously records the complete electrical waveform that carries the PD pulses using a commercial data acquisition board and extracts, in real time, the time and amplitude information of all PD pulses in software. The current approach considerably reduces the development and maintenance cost of the PD detection system, significantly increases the system portability, and may prove to be a crucial step for transferring the digital PD detection and analysis technology developed in laboratories to industry. The features of the new system are illustrated by the study of dc-excited PD pulses occurring in a point-to-plane gap in air. A new surface-mediated burst mode of PDs is discovered in which a PD pulse has a certain probability to induce another pulse. The probability is determined for several gap voltages and is found to vary strongly with the applied voltage.

## 1. Introduction

**Fig. 1:**
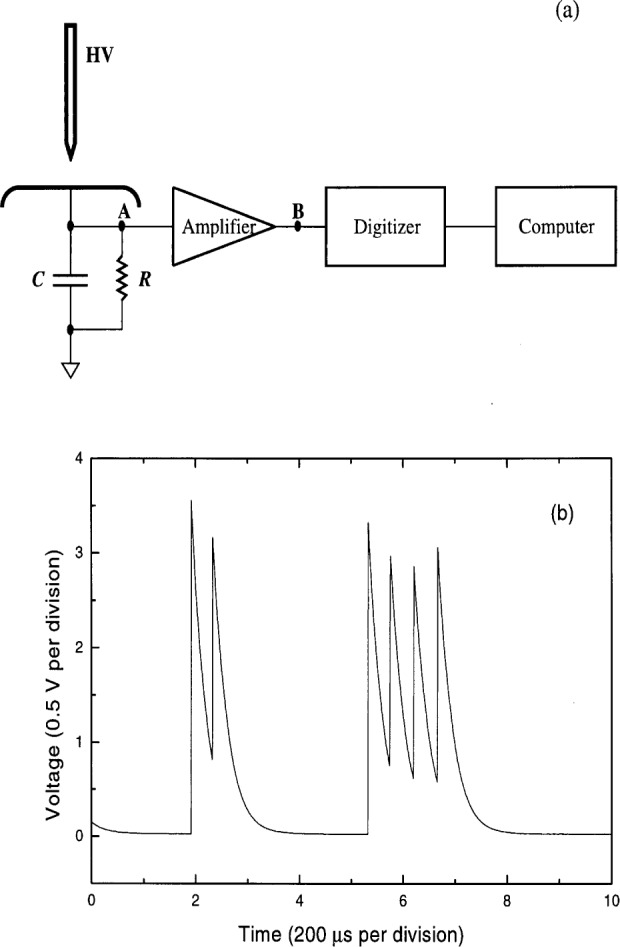
(a) Schematic diagram of the partial discharge detection system. (b) A portion of a typical long trace acquired with the system.

Partial discharge (PD) measurements are widely employed in industry for the evaluation of electrical insulation and HV apparatus [1–3]. In recent years, digital techniques for PD measurements have allowed researchers to gather a tremendous amount and variety of PD data and to envision new applications of PD measurements [4, 5]. This paper addresses a new digital method for PD detection and analysis. To better illustrate the features of the new system, let us consider dc-excited PDs that occur in a point-to-plane gap in air as an example, as shown schematically in Fig. 1.

When the voltage across the gap is sufficiently high, a pulsating PD current appears through the gap and can be detected by an external circuit. The intrinsic current pulses of the PD waveform are very narrow (≈1 ns) compared to the time separation (usually > 1 μs) between two adjacent pulses and can be attributed to the motion of the electrons created in the discharges [6]. The PD pulses form because once a discharge is initiated, the electrons are quickly depleted in the gap, either by striking the point electrode in the case of a positive PD (the point is positive with respect to the plane), or by attachment to gas-phase molecules for a negative PD. The space charge so created reduces the electric field near the point, quenches the discharge, and inhibits any new discharge until the space charge dissipates. The discharge magnitude *q*, as measured by the integrated charge of the pulse, varies from one PD pulse to another because the size of a discharge depends, in part, on the gas composition in the gap which is altered by the presence of ions, metastables, and radicals produced by the previous pulse. The size of this previous pulse, in turn, depends on the influence of its previous pulse, and so on, indefinitely. In general, the time separation from the previous pulse *t* also changes from one PD pulse to another in a non-deterministic fashion, depending on how the initiatory electrons are created and how fast the space charge is cleared.

While a PD process can be viewed as a random point process [7] and can be simply specified by a finite set of two random variables {*q_i_*, *t_i_*}, *i* = 1, 2, 3,…, the process not only defies a complete theoretical modeling but also continues to challenge experimenters for better recording systems so that the stochastic properties of PD processes can be unraveled [8]. Theoretical difficulties in dealing with partial discharges stem in part from the lack of knowledge about how a PD modifies the gas composition in the discharge gap and how the altered gas in turn influences the next PD [9]. Experimentally, there is a lack of commercial instruments that can be used to record all PD pulses in a sufficiently long period to yield statistical properties of the PD pulses.

Most digital techniques used for partial discharge measurements are limited to analyzing a few chosen parameters and cannot acquire a complete record of the PD pulse train in a given time interval. Multichannel analyzers have been used often for PD analysis, mainly for the measurement of pulse-height distribution and corresponding pulse rates [10, 11]. However, the current trend in digital PD recording and analysis is clearly to adopt computer-based measurement systems. For example, Okamoto and Tanaka [12] described a custom data acquisition system which allows for the simultaneous measurement of height and phase angle of ac-excited PD pulses. Recognizing that PD is, in general, a non-Markovian, random point process in which memory effects play an important role, Van Brunt and coworkers [13, 14] developed and reported in this journal a real-time analog PD stochastic analyzer that allows direct measurement of various conditional distributions. The analog analyzer, however, “filters” the data as received with a preset circuit and thus does not provide a complete record of all PD events during the time of observation. To remedy these limitations, von Glahn and Van Brunt [15] later designed a custom PD digitizer interfaced to a personal computer. Their digitizing system generated a complete record of all PD pulses in an indefinitely long period and subsequently allowed a complete stochastic analysis of the recorded data. Their method has revealed quantitatively, for the first time, how PD can become non-stationary even under relatively simple, well defined discharge gap conditions and has brought attention to the inadequacy of conventional PD testing standards which rely only on the apparent charge. The potential of their approach for further contributing to the understanding of the partial discharge process has been well recognized [4]. However, their PD digitizer exhibits the shortcomings of typical custom-designed fast digital circuitry, such as high development and maintenance cost, sensitivity to noise, and difficulty in keeping pace with changing personal computers. These limitations of the digitizer hinder its application in the field as a diagnostic tool for the evaluation of HV apparatus and cables.

In this paper, a different, much simpler digitizing system is described for PD measurements and analysis. The present approach digitizes the entire PD current waveform (Fig. 1) and relies on software to sort out PD pulse information {*q*_i_, *t_i_*}, while the instrument of von Glahn and Van Brunt detects each PD pulse using custom-designed hardware, with the computer serving chiefly as a storage device. The much faster personal computers and better-designed commercial data-acquisition boards now available make fast digitizing and concurrent sorting possible without any dead time. The design of the instrument is described in the next section and the capability of the new PD measurement system is illustrated with the results obtained for dc-excited partial discharges in a point-to-plane air gap in Sec. 3.

## 2. Measurement System

A simplified block diagram of the measurement system is shown in Fig. 1(a). The system was tested with partial discharge pulses occurring in a point-to-plane air gap. For such a discharge configuration, PD pulse current can be sensed and conditioned with a simple *RC* circuit in front of a high-impedance preamplifier. The values of *R* and *C* are selected so that the *RC* circuit functions as an integrator during a PD pulse, i.e., the current leaked through *R* during the PD pulse (typically < 10 ns) is negligible. For example, *C* = 1 nF and *R* = 40 kΩ were chosen for gap separations ranging from 0.5 cm to 4 cm. A typical PD waveform which appears at test point A is also shown in Fig. 1(b). Each vertical step of the waveform corresponds to a PD pulse. The 40 μs decay time is short enough to prevent overranging during pulse pile-up and long enough, compared to the sampling period (2 μs) of the recording system, for the steps to be digitized with sufficient accuracy.

The preamplifier that follows the *RC* circuit is BUF-03EJ, a high-speed monolithic voltage follower made by Analog Devices.^1^ It has an input impedance of 5 ×10^11^ Ω, an output impedance of 2 Ω, and a slew rate of 220 mV/ns.

The main hardware component of the present PD digitizing system is AT-MIO-16E-2, a multifunction data acquisition board made by National Instruments. The board is interfaced to an IBM AT-compatible computer with a 166 MHz Pentium processor, 48 Mbyte memory, and a 1 Gbyte hard disk. The data acquisition board has a sample rate of 500 × 10^3^/s and a resolution of 12 bits, with the input range programmable from ± 50 mV to ± 10 V for analog-to-digital conversion. The board is configured to continuously digitize the PD signal and cyclically fill a buffer with the acquired data. The circular buffer is configured to contain 10^6^ data points and is divided into two equal halves so that data acquired during one half can be transferred, analyzed, and saved while the other half is being filled. The system acquires 1 s long PD waveforms consecutively.

The crucial element that makes the current approach for PD measurements feasible lies in the software design. All 500 × 10^3^ data points in a just-filled half buffer have to be processed, within the 1 s period during which the other half buffer is being filled, in order to extract the time and amplitude information of all PD pulses occurring in the period. At the same time, the extracted PD data must also be transferred to the hard drive for permanent storage and further analyzed to provide basic statistical information about the acquired PD pulses, such as the distribution of amplitude and/or time separation between two adjacent pulses for on-line monitoring.

**Fig. 2:**
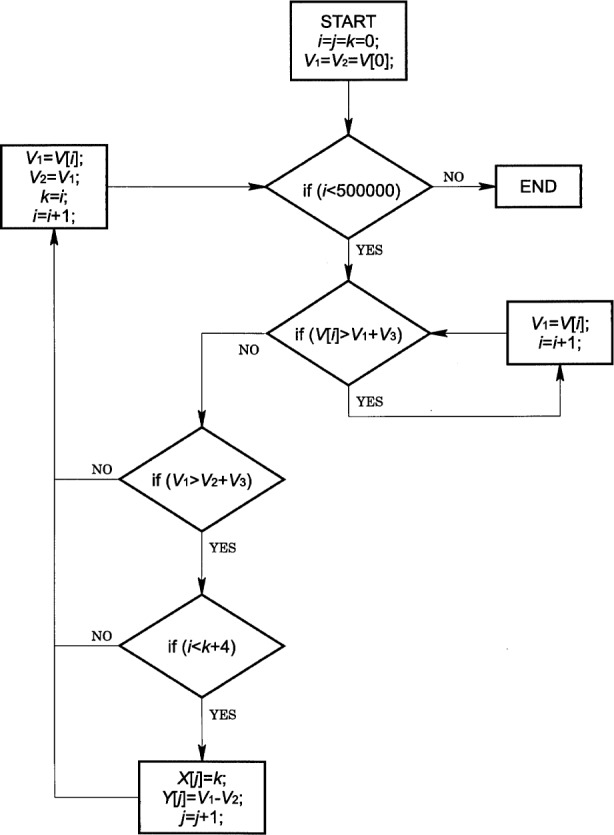
Flowchart illustrating the PD analysis algorithm; *V*_1_, *V*_2_, *i*, *j*, and *k* are local variables. The global variables *V*_3_, *V*, *X*, and *Y* are described in the text.

The time and amplitude of PD pulses are extracted from each trace with the analysis algorithm illustrated by the flowchart for positive PDs in Fig. 2.

The algorithm sorts out all vertical steps which are larger than a chosen threshold, *V*_3_, in a voltage trace stored in array *V*, and stores the amplitude and time information of each step in two arrays, *Y* and *X*, respectively. This is accomplished by letting variable *V*_1_ follow each local maximum and *V*_2_ follow each local minimum, which occurs just before *V*_1_ in time along the trace. The difference *V*_1_–*V*_2_, if above the threshold *V*_3_, is stored in the array *Y*, and the time at which *V*_2_ occurs is assigned as the PD pulse time and is stored in the corresponding element of the array *X*. Although the rise time (< 10 ns) of a voltage step corresponding to a PD pulse is much shorter than the sampling period (2 μs) of the data acquisition board, the PD step is random with respect to the sampling clock and a sample point may be taken just on the rising edge of the step. In this worst case, the PD step occupies two sampling points. Thus, the maximum time separation between *V*_1_ and *V*_2_ is 4 μs for all PD steps. This time separation is used in the analysis algorithm to reject any false steps resulting from slow-drifting noise. With this method, extraneous pulse count is essentially zero. The time resolution of the system is 6 μs, which is comparable to what can be achieved with PD detection systems using custom-designed hardware [15].

The data stored in arrays *X* and *Y* are transferred, one entire array as a block each time, to the hard disk after completing analysis of the trace. The use of block file format considerably reduces the transfer time compared to the conventional pair-wise {*X_i_*, *Y_i_*} file format. The stored data type is long integer (4 bytes) for *X* and short integer for *Y* (2 bytes). Because the number of PD pulses varies from one trace to another, the actual length of *X* and *Y* is saved as a 4 byte integer in the data file preceding *X* and *Y* for each trace. The data consecutively collected for many traces, together with other relevant parameters including the gain setting of the data acquisition board and the total input capacitance of the amplifier, can then be used off-line to reconstruct a finite set {*q_i_*, *t_i_*} which completely describes the PD activity during the period and can be subject to any conceivable statistical analysis. In recent years, researchers in the PD field have begun to explore the possibility of establishing an international standardized computer file format for storage and off-line processing of PD data [16]. While the standardization, if realized, may indeed facilitate exchanging results of PD measurements and ideas of PD analysis among various laboratories, the present work shows that a local, specialized PD data format may still be required to optimize the efficiency of PD data collection.

While continuous PD data acquisition and permanent storage are the basic components of the present PD detection system, some on-line analysis and display are also necessary for real-time utilization. The present system allows on-line calculation and display of either the amplitude distribution or the time separation distribution of PD pulses stored in *X* and *Y*. The time required to complete all the steps from acquiring trace *V* to finally displaying the calculated distribution depends on the number of PD pulses the trace carries. The maximum PD pulse rate the system can handle without missing any pulse is about 20 kHz. At this maximum pulse rate, the 1 s of computer time can be broken down as follows: (1) 0.09 s for data transfer from one half of the circular buffer to array *V*; (2) 0.36 s for sorting PD pulses from *V* to create array *X* and *Y*; (3) 0.30 s for data transfer from *X* and *Y* to the hard disk; (4) 0.23 s for on-line analysis and display of a chosen distribution; and (5) 0.02 s for waiting until the other half buffer becomes full.

Having a complete archival record of the PD data allows one to review the entire time development of PD and to make new types of analyses. The present PD measurement system has a number of off-line analysis capabilities including the calculation of first-order and second-order conditional amplitude and time-separation distributions as previously described by Van Brunt and Cernyar [13]. Some of these functions are illustrated in the next section.

## 3. Results of PD in a Point-to-Plane Air Gap

**Fig. 3:**
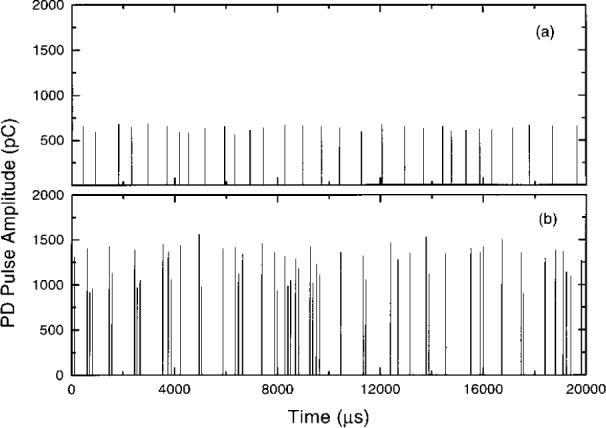
Examples of recorded PD pulses: (a) gap voltage equals 4.68 kV; (b) gap voltage equals 4.92 kV.

Examples of results obtained for positive partial discharges in a point-to-plane 5 mm air gap are presented here. The gap is formed by a 908 cone-shaped point (tip radius ≈ 50 μm) and a polished disk plane (diameter 10 cm). Both are made of stainless steel. Figure 3(a) shows a typical small portion of a long PD pulse train recorded during a period of 300 s at an applied gap voltage of 4.68 kV. As can be seen in the figure, both the pulse amplitude and the time separation between two adjacent pulses vary. The variability results in part from where and when the seed electron that initiates an electron avalanche appears. If the seed electron appears too far from the stressed electrode in a region where attachment dominates, the electron avalanche cannot form. If the electron is too close to the electrode, on the other hand, the avalanche strikes the electrode before it can reach the critical size, which is about 10^8^ electrons, to form a detectable PD pulse [17]. The seed electrons are delivered by negative ions such as $O_{2}^{-}$ and some hydrated ions which result from the background radiation in the atmosphere. A negative ion can detach its extra electron through a collision near the point electrode where the local electric field is sufficiently high [18].

**Fig. 4:**
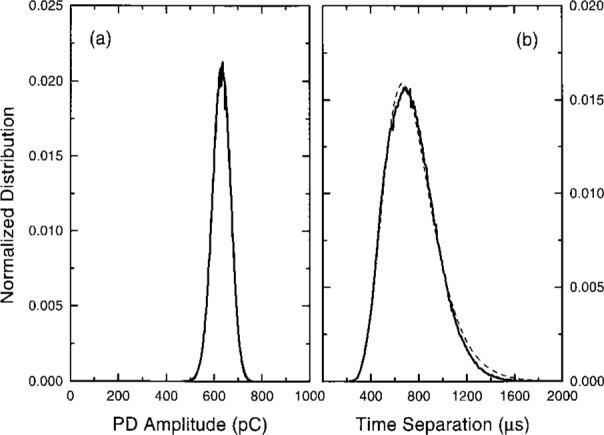
(a) PD pulse amplitude distribution. The dashed line, which is fitted to a Gaussian function, overlaps completely with the solid line representing the experimental data. (b) Distribution of time separations. The dashed line results from a least-squares fit to a log-normal distribution function. Both (a) and (b) are normalized to unit area and are the results from the same data file acquired at a gap voltage of 4.68 kV.

Figure 4(a) shows the PD pulse amplitude distribution corresponding to Fig. 3(a). The distribution has a Gaussian shape; a curve fitted to a Gaussian function overlaps completely with the solid line representing the data. This characteristic has been observed earlier by Malik and Al-rainy [11] using a multichannel analyzer under similar conditions. The present system, however, allows other statistical analyses at the same time. Figure 4(b) shows the distribution of the time separation between two adjacent pulses for the same PD data file. The distribution closely follows a log-normal distribution function of the form:

$$Pdt=\frac{1}{\sqrt{2\pi \sigma t}}\exp\left[-\frac{\ln^{2}(t/t_{c})}{2\sigma^{2}}\right]dt$$ (1)

as indicated by the least-squares fitted curve in the figure. A transformation gives a normal distribution with fitting parameters *σ*, and *χ*_c_ ≡ ln *t*_c_. This type of distribution has also been observed in the time-lag experiments of Berger et al. [19], where the time lag of the first PD pulse is measured after applying a step voltage across the gap at time *t* = 0. There is no satisfactory explanation yet for the physical origin of the observed log-normal distribution. This is in contrast with the well understood normal distribution [20], such as the amplitude distribution [Fig. 4(a)] which results cumulatively from many independent factors including the distribution of the space charge, the location where the seed electron appears, and the structure of the electron avalanche created, etc.

When the gap voltage is raised, a previously unreported burst mode of partial discharge appears, as shown in Fig. 3(b) which was recorded at 4.92 kV for this experimental setup. As can be seen in the figure, the amplitudes of the PD pulses have almost doubled compared to those at 4.68 kV, and the pulse rate also increases. The rate increase is mainly due to the formation of bursts where one pulse tends to be followed closely by another. The pulses in a burst are almost evenly separated, which is different from the PD burst pulses observed previously [21] in SF_6_ that have irregular time separations. The burst mode observed here is strongly affected by the cathode surface condition: the bursts disappear when the polished stainless steel cathode is replaced by an unpolished stainless steel electrode, with all other conditions maintained the same. This is in contrast with the non-burst mode at lower voltages where replacing the electrode has no observable effect on PD. A possible explanation is that the pulses following the leading pulse of a burst are initiated by the negative ions that are produced on the cathode surface. The negative ions can be produced either directly through negative-ion desorption induced by photons [22] and/or positive ions [23] that are created in a PD pulse, or indirectly through secondary electron emission and subsequent electron attachment [24] near the surface. All these secondary processes are very sensitive to the surface condition and are probably not efficient enough to have any observable effects on PD for an unpolished cathode whose surface is covered by a thick oxide layer.

**Fig. 5:**
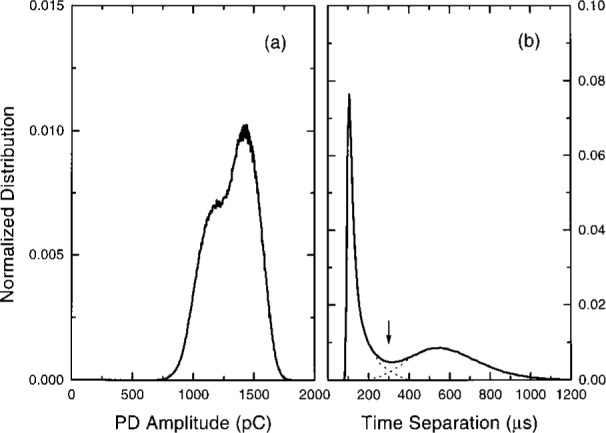
(a) PD pulse amplitude distribution. (b) Distribution of time separations. The dashed lines indicate the overlap between the two peaks. Both (a) and (b) are normalized to unit area and are the results from the same data file acquired at a gap voltage of 4.92 kV.

Figure 5(a) shows the PD pulse amplitude distribution recorded in a 300 s period at 4.92 kV which exhibits “burst” behavior. This distribution is wider compared to the amplitude distribution recorded at 4.68 kV and appears to be a superposition of two or more Gaussian-like peaks. The distribution of time separation shown in Fig. 5(b) forms a two-peak pattern, with a narrow peak around 100 μs in addition to the broad peak which is similar to that observed in the non-burst mode. While most patterns observed in PD distributions cannot be readily interpreted [25, 26], the simple patterns observed in this study are clearly attributable to the bursts. More laboratory work on this type of understandable pattern will undoubtedly help improve and facilitate the use of pattern recognition for diagnosing high-voltage apparatus.

**Fig. 6:**
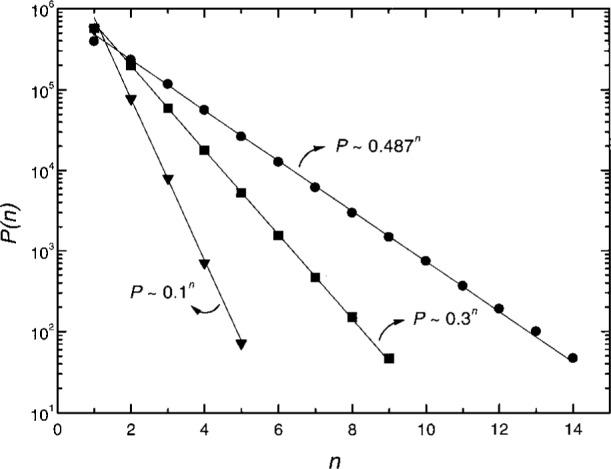
Total number *P*(*n*) of *n*th pulses accumulated over a period of 300 s as a function of pulse index number *n* (*n* = 1 for the first pulse in a burst, *n* = 2 for the second pulse, etc.). Solid circles are for a gap voltage of 4.92 kV, and solid squares are for a gap voltage of 4.84 kV. Solid triangles are for a gap voltage of 4.74 kV.

While each PD pulse in a burst except the first is induced by its corresponding previous pulse, not all pulses can bring in a follower. If they could, the burst length, measured by the number of pulses in a burst, would not be finite, as seen in Fig. 3(b). An interesting question is then: “What is the probability *p* for a PD pulse in a burst to induce a following pulse, and does such a probability depend on the pulse index number, *n* (*n* = 1 for the first pulse in a burst, *n* = 2 for the second pulse, etc.)?” To address this question, we need a clear method to distinguish bursts. Most bursts are well separated in time but a few bursts are very close to each other [Fig. 3(b)]. This is manifested by the non-zero overlap between the two peaks in the time separation distribution in Fig. 5(b). The broad peak results from the PD pulses initiated by the background negative ions while the narrow peak results from the surface-enhanced PD. If we take the minimum (*t* = 300 μs) between the two peaks as the critical separation for the two PD initiating modes, the resultant error in distinguishing bursts will be about 5 % as suggested by the dashed lines in Fig. 5(b). With this separation method, we can then analyze all the bursts that were recorded at 4.92 kV. Shown in Fig. 6 is the accumulated number, *P*(*n*), of *n*th pulses with *n* ranging from 1 to 14. The data of *P* vs *n* fall in a straight line in the semi-log plot, i.e., the probability does not depend on the pulse index number. A fit to *P*(*n*) ~ *p^n^* yields *p* = 0.487 for the gap voltage of 4.92 kV. The probability decreases when the gap voltage is lowered with *p* = 0.3 at 4.84 kV, *p* = 0.1 at 4.74 kV, and *p* = 0 at 4.68 kV.

**Fig. 7:**
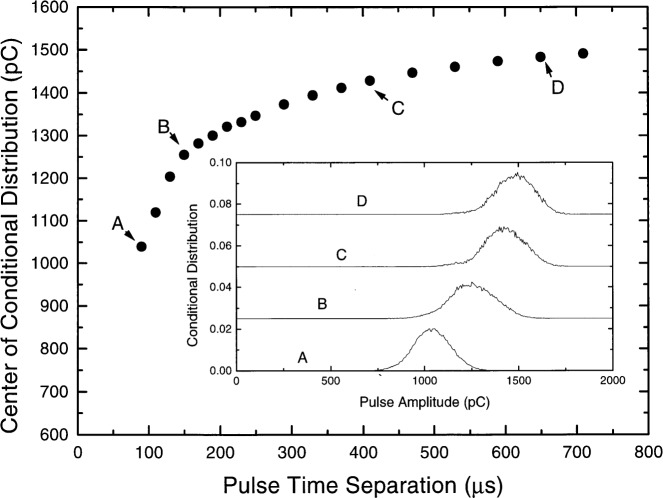
Center of the first-order conditional amplitude distribution as a function of pulse time separation. The inset shows the detailed conditional distributions for time separations indicated by the labels A, B, C, and D.

The influence of the space charge produced in a PD pulse on the amplitude of its subsequent PD pulse can also be analyzed from the recorded data. It is generally accepted that partial discharge is pulsating because of the space charge created by the discharge. The space charge reduces the local electric field near the point electrode to a level that eventually quenches the discharge. The space charge also inhibits the next pulsating discharge until the space charge moves away [6]. However, how long the space charge can keep inhibiting the next PD cannot be clearly described by a single number. The space charge occupies a certain volume and contains a variety of ionic species which in general have different mobilities, and the next PD pulse may occur before the space charge completely dissipates. The influence of the residual space charge on the PD pulse amplitude is illustrated in Fig. (7), where the first-order conditional pulse amplitude distributions *p*_1_(*q_i_*|*t_i_* ± Δ*t*) are shown in the inset. Here *p*_1_ is the probability that the *i*th pulse has an amplitude in the range of *q_i_* to *q_i_* + d*q* if its time separation from the previous pulse has a value in the range *t_i_* ± Δ*t*, and Δ*t* = 10 μs was chosen for all the conditional distributions analyzed. As can be seen in the inset of Fig. (7), the conditional amplitude distributions have a Gaussian shape. The center of the distribution shifts consistently towards higher amplitudes as *t_i_* increases from 90 μs to 150 μs, 410 μs, and 650 μs. A plot of the distribution center, resulting from a least-squares fit, vs *t_i_* is shown in Fig. (7). The curve rises rapidly with increasing *t_i_* for *t_i_* < ≈150 μs and then slowly levels off, which is consistent with the fact that the space charge continuously dissipates and moves away from the region near the point electrode where it has more influence on the size of the next PD pulse.

## 4. Conclusions

It is demonstrated that the new PD detection system, which relies on computer software to extract PD pulse and time information, provides the capability to record continuously all the information about PD pulse amplitude and time over extended periods of time. The detection system has a minimum inter-pulse time resolution of 6 μs and a vertical amplitude resolution of 12 bits. Having a complete archival record of the PD data allows one to review the entire time development of PD and to make new types of analyses.

Analysis of the positive PD data acquired for a point-to-plane air gap reveals a new burst mode of PD in which each PD pulse has a fixed probability to induce another pulse, forming a burst of almost evenly-spaced pulses. This burst mode is probably mediated by the cathode surface acting as an extra source of negative ions. The negative ions can be produced from the surface either directly through negative-ion desorption induced by photons and/or positive ions that are created in a PD pulse, or indirectly through secondary electron emission and subsequent electron attachment near the surface. These surface-born negative ions drift towards the point electrode and may provide a seed electron to initiate another PD pulse via collisional detachment [18] in the region where the local electric field is sufficiently high.

## Footnotes

1 Certain commercial equipment, instruments, or materials are identified in this paper to foster understanding. Such identification does not imply recommendation or endorsement by the National Institute of Standards and Technology, nor does it imply that the materials or equipment identified are necessarily the best available for the purpose.
